# Specific bacterial microbiome enhances the sexual reproduction and auxospore production of the marine diatom, *Odontella*

**DOI:** 10.1371/journal.pone.0276305

**Published:** 2022-10-19

**Authors:** Marilou P. Sison-Mangus, Michael W. Kempnich, Monica Appiano, Sanjin Mehic, Terril Yazzie

**Affiliations:** Department of Ocean Sciences and Institute of Marine Sciences, University of California, Santa Cruz, Santa Cruz, California, United States of America; CSIR-National Institute of Oceanography, INDIA

## Abstract

Auxospore production is a sexual reproductive strategy by diatoms to re-attain normal size after the size-reducing effect of clonal reproduction. Aside from the minimum size threshold used as a sex clock by diatoms, the environmental or chemical triggers that can induce sex in diatoms are still not well understood. Here we investigated the influence of six marine bacteria from five families on the production of sexual cells and auxospores of the ubiquitous marine polar centric diatom, *Odontella* sp. Microbiome association and co-occurrence with the diatom in culture and in nature were investigated using 16S rRNA amplicon sequencing. Indole acetic acid (IAA) secretion, a phytohormone that regulates plants’ growth and sexual development, was explored as a potential inducer of sexual reproduction in *Odontella* and compared between bacterial associates. We found that *Odontella* co-cultured with *Flavobacteriaceae* (*Polaribacter and Cellulophaga*) have significantly more sexual cells and auxospores than bacteria-free *Odontella* and *Odontella* co-cultured with other bacteria from *Vibrionaceae* (*Vibrio*), *Pseudoalteromonadaceae* (*Pseudoalteromonas*), *Rhodobacteraceae* (*Sulfitobacter*), or *Planococcaceae* (*Planococcus*) family. Differences in IAA secretion were observed between bacterial isolates, but this did not correspond consistently with the diatom’s clonal growth or production of sexual cells and auxospores. Microbiome composition survey of *Odontella* cultures showed that the diatom harbors homologous sequences of the four bacterial isolates at varying proportions, with *Sulfitobacter* and *Polaribacter* at high abundances. Microbiome surveys at Santa Cruz Wharf, Monterey Bay, from 2014–2015 showed that *Odontella* abundance is positively correlated with *Flavobacteriaceae* and *Rhodobacteraceae* abundances. Our study demonstrates that specific members of the diatom microbiome can enhance the host’s sexual reproduction, with the interkingdom interaction driven by partner compatibility and long-term association. Sex-enhancing bacteria may even be needed by the diatom host to carry out the optimal inducement of sex under normal conditions, allowing for size restitution and maintaining genetic diversity in culture and in nature.

## Introduction

Marine diatoms are one of the major phytoplankton groups that support the ocean’s biological productivity and are responsible for 20% to 40% of the primary production on Earth [1]. Diatoms tend to dominate the phytoplankton assemblage in the coastal oceans with upwelling features and create massive blooms when conditions are favorable. Such blooms depend on the rapid asexual reproduction of diatoms through mitosis [2], which facilitates their rapid growth and biomass accumulation. However, each round of mitosis requires that each daughter cell rebuilds half of its frustule in a process that decreases the average physical size of the dividing clonal cell [3]. Eventually, the diatom cell reaches a critical minimum size which triggers the cell to undergo sexual reproduction to restore its maximal cell size via auxosporulation [4]. Genetic variants are also introduced in the population via sexual reproduction, which increases the organism’s potential for evolutionary adaptations in a changing environment.

The critical minimum size or the diatom sex clock [5] differs between diatom groups, with various species initiating sexual reproduction at 40% -71% of their maximum size [4, 6, 7]. Upon reaching the critical size threshold and receiving a specific environmental trigger, the cell produces gametes with the viable zygote developing into an auxospore. Pennate and centric diatoms vary regarding the production of gametes. Pennate diatoms are heterothallic, producing M- and M+ mating types [8]. A pheromone guides the pairing and fusion of the two mating types, forming a zygote, which then develops into an auxospore [9, 10]. On the other hand, centric diatoms are homothallic, producing differentiated gametes with the sperm swimming towards the eggs during fusion. The auxospore is then extruded and developed into a large initial cell.

Abiotic and biotic factors can influence diatom sexual reproduction. The nutrient ammonia, along with phosphorous or silica limitation, can trigger sexual reproduction in *Thalassiosira pseudonana* [11], while low light and short photoperiod can induce sexual reproduction in the pennate diatom *Haslea ostrearia* [12]. Biotic factors such as associated bacteria can also play a role in stimulating or inhibiting the sexual reproduction of a benthic, pennate diatom, *Seminavis robusta* [13]. The microbiota can modulate the diatom’s synthesis of di-L-prolyl diketopiperazine (diproline), a sex pheromone that induces sexual reproduction in microalgae [9, 14]. Indeed, the environmental factors or chemical cues that trigger the inducement of sexual reproduction and auxosporulation in marine diatoms are complex and not well understood [15].

The influence of marine bacteria on phytoplankton biology and ecology was recognized with the observations that bacterial productivity synchronizes with phytoplankton productivity [16]. Co-occurrence between phytoplankton and specific bacterial groups has also been observed during algal blooms [17–20]. Although the relationship between phytoplankton and bacteria tends to center on microbial transformations of phytoplankton biomass via particulate degradation and remineralization [21, 22], recent works have highlighted their role in sustaining phytoplankton growth directly. For instance, the conversion by associated bacteria of diatom-released tryptophan to indole acetic acid [23] and the bacterial release of vitamins directly benefit the auxotrophic microalgal partner [24, 25]. Some associated bacteria can enhance the production of diatom neurotoxin [26, 27], while other bacteria can aid the cyst formation of dinoflagellates [28]; the metabolic mechanisms for these latter processes, however, are unknown.

Besides being ecologically important, growing interest in understanding diatom biology stems from their promising potential for nanotechnology, medicine, biofuel production, and pharmaceutical products (reviewed in [29]). Knowledge of the factors that could induce the diatom’s sexual reproduction can improve the genetic manipulations of its commercially desirable traits and the potential for long-term maintenance in culture; both are valuable tools for the sustainable industrial use of diatoms. In this study, we investigated the influence of six phytoplankton-associated bacteria on the growth and sexual reproduction of the ubiquitous marine diatom, *Odontella sp*., a polar-centric planktonic diatom that is commonly found in the coastal environment. Its congeneric, *Odontella aurita*, is cultured commercially for its production of polyunsaturated fatty acids and is used for human health and nutrition [30]. The six bacteria used in the study belong to five bacterial families (*Rhodobacteraceae*, *Flavobacteriaceae*, *Pseudoalteromonadaceae*, *Vibrionaceae*, and *Planococcaceae*) that are often reported to be associated with phytoplankton blooms. The co-occurrence of these bacterial families with the *Odontella* population in the coastal environment was assessed for two years (2014–2015). In addition, the microbiome of four *Odontella* cultures isolated initially from within this sampling time frame was examined after 130 culture generations to determine the stability of the microbial associations. Bacterial secretion of IAA was measured in the absence and presence of tryptophan, an amino acid precursor for IAA biosynthesis that is typically released by phytoplankton as exudate. Our results suggest that only certain bacteria can enhance the sexual reproduction of *Odontella*, and these sex-promoting bacteria tend to co-occur with the diatom in nature and persist under diatom culture conditions.

## Materials and methods

### Microbiome and phytoplankton sampling

From April 2014 to December 2015, weekly water sampling for the bacterial community assemblage was done at Santa Cruz (SC) Wharf, Monterey Bay, California (36°57’28.6”N 122°01’04.1”W) using a Niskin bottle, collecting water at a depth of 0, 1.5 and 10 meters. The detailed sampling method and the methods for sample processing, DNA extraction, sequencing, and analysis are described in [31]. Briefly, homogenous water samples were filtered with 3.0 μm Durapore filter membrane (Millipore, USA), and DNA was extracted using MoBio Kit (USA). Equimolar concentration was sent to Argonne National Laboratory Sequencing Facility. 16S rRNA was paired-end sequenced with 515F-806R universal primers (5’GTGCCAGCMGCCGCGGTAA 3’ and 5’GGACTACHVGGGTWTCTAAT 3’, respectively) using Illumina Miseq Sequencing platform [32]. Sequences were then processed using the QIIME 1.91 pipeline [33]. The 16S rRNA sequences were deposited under BioProject # PRJNA648155 with respective BioSample IDs in S1 Table.

Data for the relative abundance of *Odontella* genus in SC Wharf was taken from weekly phytoplankton assemblage reports of the California HabMap Project (http://oceandatacenter.ucsc.edu/PhytoBlog/) for the same years. Phytoplankton abundance was assessed via microscopy using qualitative measures and were translated to average values to determine relative abundance of *Odontella* in the phytoplankton assembly: None: 0; Rare: <1% (0.5); Present: 1–9% (5%); Common: 10–49% (30%) and Abundant: > = 50%-100% (75%).

### *Odontella* microbiome survey using 16S rRNA sequencing

Four *Odontella* cultures were isolated from single cells on May 22–28, 2014, from net tows taken from SC Wharf, California, and maintained for five years (~130 generations) in L1 medium until the experiment. The algae were subcultured every 10–12 days by transferring 200 μl of algal inoculum to new L1 media that was filtered (0.2 μm) and autoclaved. Their associated microbiome was harvested by filtering *Odontella* cells in 5 μm Durapore membrane and stored at -80°C in December 2020. DNA extraction and 16S rRNA sequencing was carried out as above and sequences were deposited under BioProject # PRJNA648155 with respective Biosample IDs in S1 Table.

### Bacterial isolation and genotyping

Membrane-filtered seawater samples taken from SC Wharf on May 2014 were dabbed in marine agar 2216 (BD Difco, USA) and grown at room temperature for several days to isolate phytoplankton-associated bacteria. Individual bacterial colonies with different morphotypes were streaked repeatedly in new agar plates until pure single strains were isolated. Each pure strain was grown in marine broth 2216 (BD Difco, USA) at room temperature, and an aliquot was mixed with autoclaved 50% glycerol for storage at -80°C.

Each strain was genotyped by amplifying the partial 16S rRNA via PCR using the universal primers 8F and 1492R [34] under the following PCR condition: 35 cycles of 95°C for 30 sec, 50°C for 30 sec, 72°C for 2 min, and a final extension step at 72°C for 5 min. An aliquot of the PCR product was sent to UC Berkeley sequencing facility after confirming the presence of a single 16S PCR band product in 1% agarose gel. Sequences were viewed in Geneious 10.2 software and manually checked for base-calling accuracy and were named accordingly by their homologous 16S rRNA sequence in GenBank using BLAST [35].

The 16S RNA sequences of the bacterial isolates chosen for the experiments below were deposited in NCBI with the following GenBank Numbers: MSM-37_PP-B3A.1B (MW089308, *Polaribacter* sp. belonging to *Flavobacteriaceae* family), MSM-36_SCFSW8 (MW089307, *Cellulophaga* sp., belonging to *Flavobacteriaceae* family), MSM-2_PP-B2A.1 (MW089306, *Planococcus* sp., belonging to *Planococcaceae* family), MSM-74_PN4-2A.30 (MW089310, *Pseudoalteromonas* sp, belonging to *Pseudoalteromonadaceae* family), MSM-75_PN4-2B.31 (MW089311, *Vibrio* sp., belonging to *Vibrionaceae* family) and MSM-66_SC77-PNM (MW089309, *Sulfitobacter* sp., belonging to *Roseobacteraceae* family).

The phylogenetic relationship of the bacterial isolates to other marine bacteria was constructed using 16S rRNA sequences, and the tree was made along with other homologous bacterial sequences downloaded from GenBank. The Neighbor-Joining method [36] with Jukes-Cantor as the model of DNA evolution and 1000 bootstrap for tree robustness analysis were used in building the phylogenetic tree in Geneious 10.2 software.

### Scanning Electron Microscopy (SEM)

Bacteria isolates were cultured in Marine Broth 2216 (Difco, USA) overnight and harvested via centrifugation at 3000 g for 5 min. Bacterial pellets were resuspended in 3-ml autoclaved, 0.2 μm-filtered seawater (FSW) with an OD_600_ of 0.3 and were gently filtered onto sterile 0.2 μm black polycarbonate filters (Millipore, USA) and washed twice with FSW. A freshly made 3% glutaraldehyde in PBS solution was added to the membrane filters for a fixation of 1 hour. Filters were briefly washed with 1X PBS and dehydrated in graded ethanol series (20, 50, 70, and 100%) for 10 min. Samples in filter membranes were stored in 100% ethanol before microscopy. At the UC Santa Cruz SEM facility, samples were critical-point dried, gold-coated with 20-nm gold particles, and imaged with FEI Quanta three-dimensional (3D) dual-beam microscope.

### Phytoplankton- bacteria co-cultures

*Odontella sp*. clone #4 was made bacteria-free by growing the culture in L1 media with Ampicillin (1 mg/ml) and Kanamycin (10 μg/ml) over two growth cycles of 7 days each. At the end of the second growth cycle, the culture was assessed for axenicity via 1) sterility test by growing in Difco Marine Broth 2216 and Difco Marine Agar (BD, USA) for two days and 2) amplification of 16S rRNA via PCR using universal bacterial 16S rRNA primer 8F and 907R (5′-CCGTCAATTCMTTTRAGTT-3′) using the PCR program mentioned above. The bacterial medium inoculated with antibiotic-treated algae was clear after two days. No gene product was seen after 16S rRNA PCR amplification, suggesting that both tests did not detect bacteria in the *Odontella* culture. The generation of bacteria-free phytoplankton is further described in [26].

**Fig 1:**
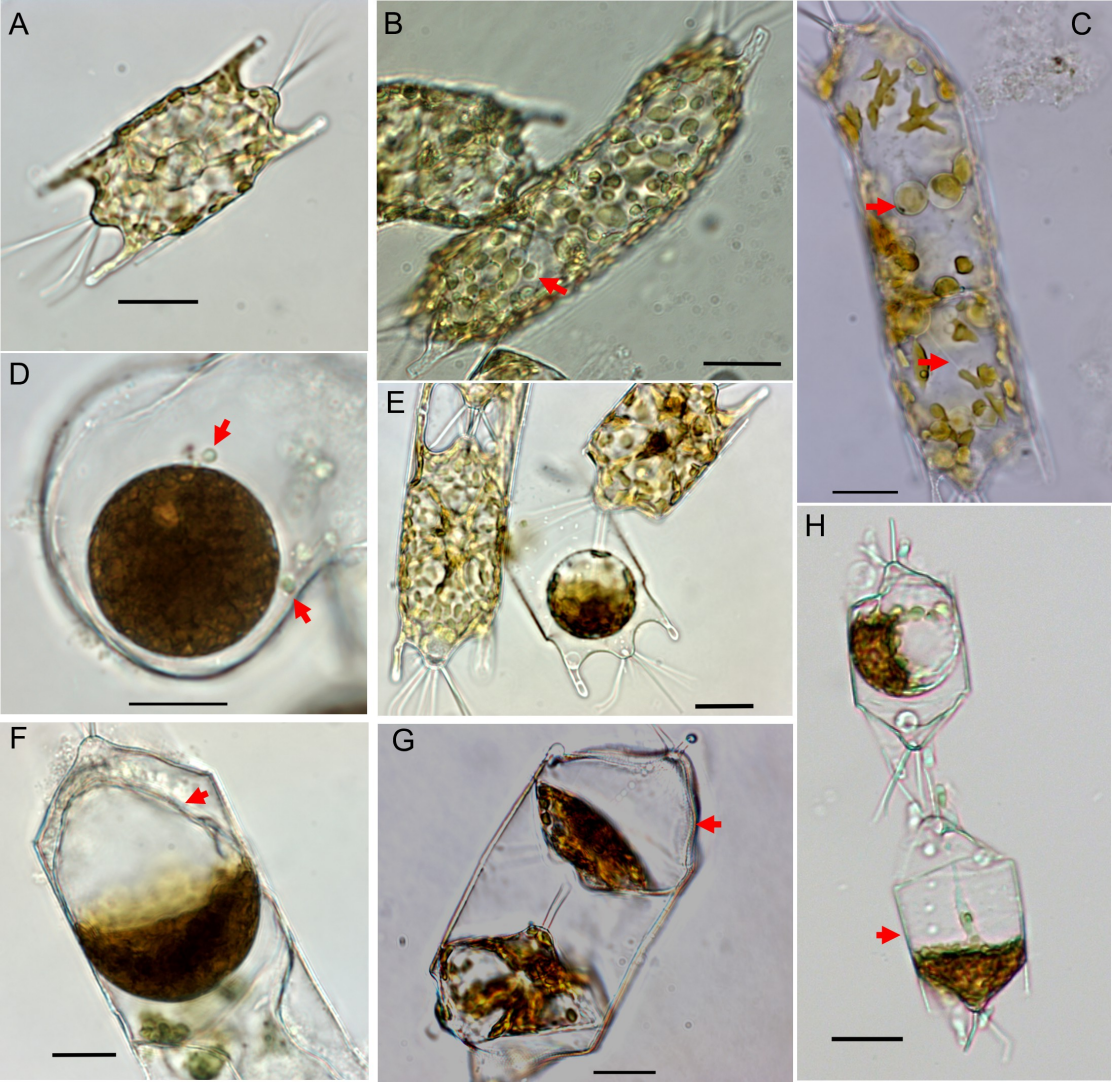
Reproductive stages and the auxospores of *Odontella* sp. diatom. (A) Vegetative *Odontella* cell. (B) Spermatogonium with primary spermatocytes (arrow) (C) Spermatocytes (arrow) and sperm cell (arrow pointing to its tail) inside the spermatogonium. See S1 Video displaying the movement of the tail. (D-E) Oogonium, the two arrows point to sperms attached to the egg in D. (F-H) Auxospores, arrow points to the properizonium. Black scale bar is 20 μm.

Axenic *Odontella sp*. was grown in L1 media [37] on a 16:8 day-night cycle with a daytime light incidence of ~ 100 μmol photons m^-2^ s^-1^ at 15–16°C temperature range (hereafter “phytoplankton growth conditions”). The bacteria were grown for two days in Difco Marine Broth 2216 at ambient temperature, washed via centrifugation at 3000 g for 5 min, and resuspended in L1 media. OD_600_ of each bacterial suspension was between 0.9–1.0 at a bacteria cell density range of 2.7–2.9 x 10^6^ cells/ml. Bacterial cell density was counted using Amnis FlowSight cytometer (AMNIS, USA) after fixing the aliquot sample in 1% paraformaldehyde. For the experiment, 50-ml L1 media was inoculated with exponentially-growing axenic *Odontella* at a cell density of 408 ± 6 cells/ml and co-cultured with different bacteria: *Polaribacter* sp. *(*MSM-37_PP-B3A.1B strain*)*, *Sulfitobacter* sp. (MSM-66_SC77-PNM strain), *Vibrio* sp. (MSM-75_PN4-2B.31 strain), *Cellulophaga* sp. (MSM-36_SCFSW8 strain), *Pseudoalteromonas* sp. (MSM-74_PN4-2A.30 strain), or *Planococcus* sp. (MSM-2_PP-B2A.1 strain), while one treatment set was grown as bacteria-free *Odontella* culture and serve as a control. The experimental cultures were grown under phytoplankton growth conditions for 18 days, with measurements taken on days 5, 8, 12, 15, and 18 for bacteria density, *Odontella* cell density, *Odontella* undergoing sexual reproduction (sexual cells), and auxospores. Fig 1 shows images of respective *Odontella* cells tracked in the study.

### Algal cell density and auxospore counting

Samples of each *Odontella* culture were counted using light microscopy for algal cell density and the presence of auxospores and other sexual reproductive stages. An aliquot of 1.8 ml *Odontella* culture was preserved and stained by adding 0.2 ml Lugol’s solution. Algal samples and bacterial counts were taken on days 5, 8, 12, 15, and at day 18 when cultures start to reach the stationary phase. Preserved aliquot samples were counted using a Sedgewick-Rafter counting chamber. From a grid of 1000 1μL squares, 20 squares were randomly picked when counting phytoplankton and sexual cells, while 280 squares were picked to count auxospores. Growth rate was calculated at the log phase of growth, between Day 7 and Day 15 using the following equation: μ = ln(N_2_/N_1_)/ (t_2_ − t_1_), where μ is the specific growth rate, and N_1_ and N_2_ are cell densities at time 1 (t_1_) and time 2 (t_2_), respectively.

### Algal growth and sexual reproduction with IAA

The effect of IAA on diatom growth and auxospore production was also tested by exposing axenic *Odontella* to the chemical IAA (Sigma, USA) for 18 days to determine if IAA alone can enhance the growth and auxospore production of bacteria-free *Odontella*. The same experimental procedure as described above for diatom-bacteria co-cultures was applied. In the experimental treatment, 0.2 μm filter-sterilized IAA was added to 50-ml sterile L1 media at 50 nM concentration (n = 5), while the control medium was left unamended with IAA (n = 5). Both media were inoculated with bacteria-free *Odontella* with a similar inoculum algal density of 290 ± 39 cells/ml and incubated in similar phytoplankton growth conditions for 18 days. Counts were made starting on Day 7 and carried out every four days until Day 18.

### Indole-3-acetic acid (IAA) measurement

The six bacteria used in the above experiment were grown in triplicate cultures in 25% strength Difco Marine Broth 2216 with NaCl concentration maintained to 19.45g/L with no tryptophan and with tryptophan added at 0.1% w/v. Cultures were grown in a shaker at ambient temperature to exponential phase (2 days). LB broth only and LB with tryptophan were used as negative controls in the experiment. An aliquot of 500μL was fixed with 1% paraformaldehyde for bacterial counting using imaging flow cytometry. The remaining culture was centrifuged at 3000g for 5 min, and the supernatant was taken to measure extracellular IAA concentration through reaction with Salkowski reagent. A standard curve was created using IAA at concentrations from 0–100 μg/mL in 25% Difco Marine Broth. A 100 μl volume of each standard in triplicate and each sample in duplicate were mixed with 200 μl Salkowski reagent in a 96-well plate and were incubated for 30 min before absorbance was read at 530nm using a Victor X3 Multimode Plate Reader (Perkin Elmer, USA). LB broth only and LB with tryptophan were used as negative controls in the measurement.

### Statistical analysis

Algal cell density, number of sexual cells, number of auxospores, and IAA concentration between treatments were log-transformed and compared for statistical differences using one-way ANOVA followed by Tukey-HSD posthoc test, after satisfying the conditions of normality and Bartletts’ test of homogeneity of variance. The density-normalized abundance of auxospores and sexual cells were arcsine-transformed before performing ANOVA. Algal growth rates between treatments were statistically compared using the Kruskal- Wallis sums rank test, followed by mean comparisons using Wilcoxon pair test. Data from the *Odontella* growth experiment amended with IAA were analyzed using paired T-test. Correlations between bacterial and *Odontella* populations were estimated using the Spearman ranks test. Data used for the correlational analyses were log-transformed into centered log-ratio (clr) values using the cmultRepl command from the zCompositions R package [38] to determine the abundance of OTUs relative to the per-sample average (geometric mean). A detailed description of the compositional data analysis for the bacterial OTU data is reported in [31]. All statistical analyses were conducted using Graphpad Prism 9.0.2 statistical software, except for the compositional data analysis and clr data transformation of the bacterial OTUs abundance, which was carried out in R [39] (S1 File).

## Results

### *Odontella* sexual cells and auxospore morphology

The *Odontella* sp. used in the study is a polar-centric diatom with a varied size between 60–110 μm with two long tapering horns and a cone in the middle topped with two long divergent spines (Fig 1A). The diatom is homothallic, capable of producing anisogamous gametes. The cell undergoes spermatogenesis resulting in cell elongation during the production of sperm (Fig 1B and 1C, S1 Fig). During oogenesis, a diatom cell is observed to produce a single or two oogonia (Fig 1D and 1E, S2A–S2F Fig). During release, a hump or an opening form in the frustule after plasmolysis, and an egg cell protrudes from the cell or is released in the water (S2C, S2D and S2F Fig). Fertilization occurs when the sperm and egg cells meet (Fig 1D), and nuclear content is transferred. An auxospore develops after fertilization, resulting in a large circular or semi-conical cell (Fig 1F–1H, S2H–S2L Fig). A specific description of the process of sexual reproduction in *Odontella regia* and the related species *Biddulphia tridens* were reported in Hegde et al. [40] and Samanta et al. [41], respectively. Von Stosch [42] *Biddulphia rhombus* auxospore images were used as an additional reference for our auxospore images.

### Bacteria morphology and phylogeny

**Fig 2:**
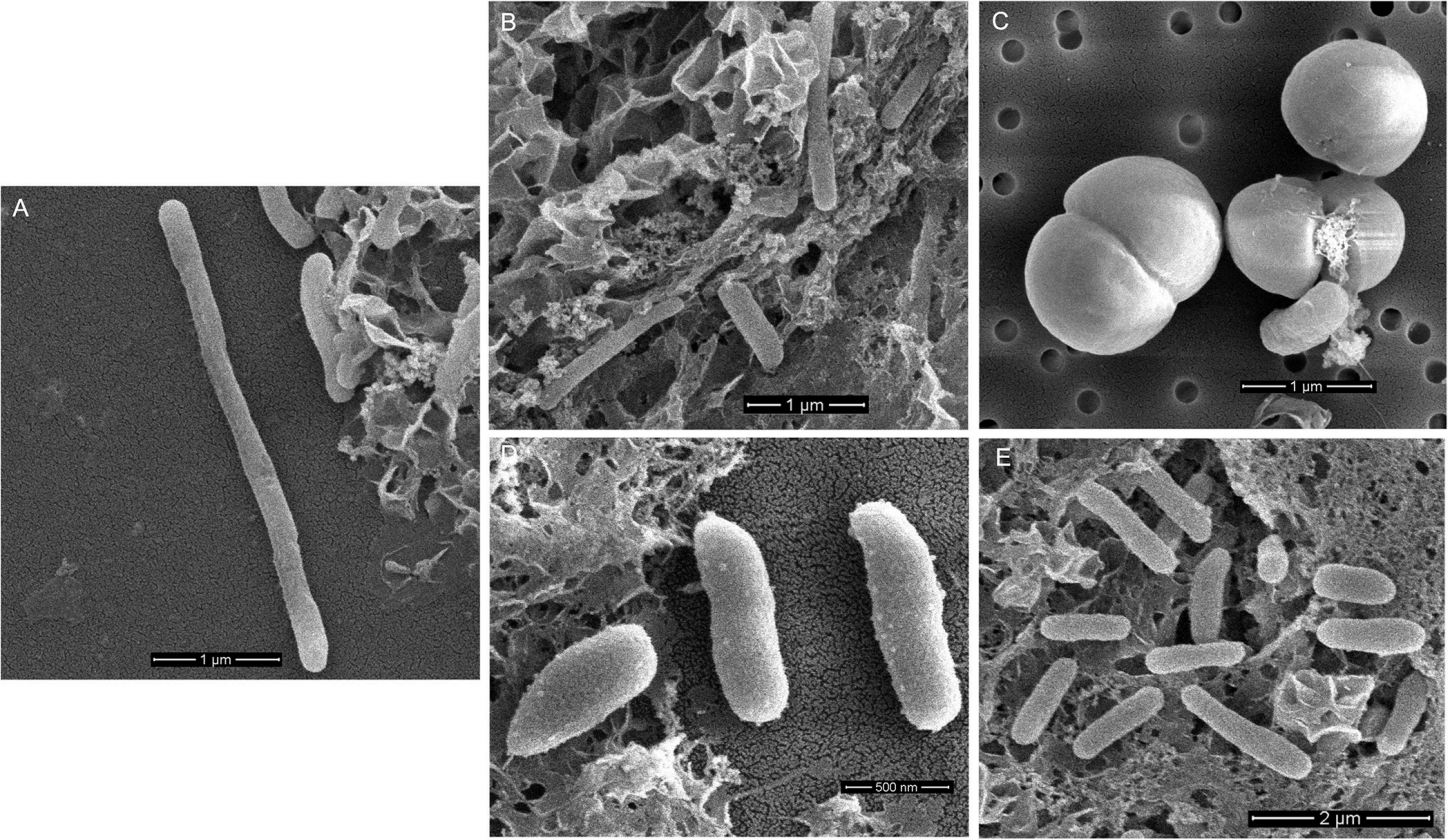
Scanning electron micrographs of isolated bacteria used in the experiment. **(**A) *Cellulophaga* sp. (Cel) (B) *Polaribacter sp*. (Pol) (C) *Planococcus* sp. (Plan) (D) *Pseudoalteromonas* sp. (Palt) (E) *Vibrio* sp. (Vib).

**Fig 3:**
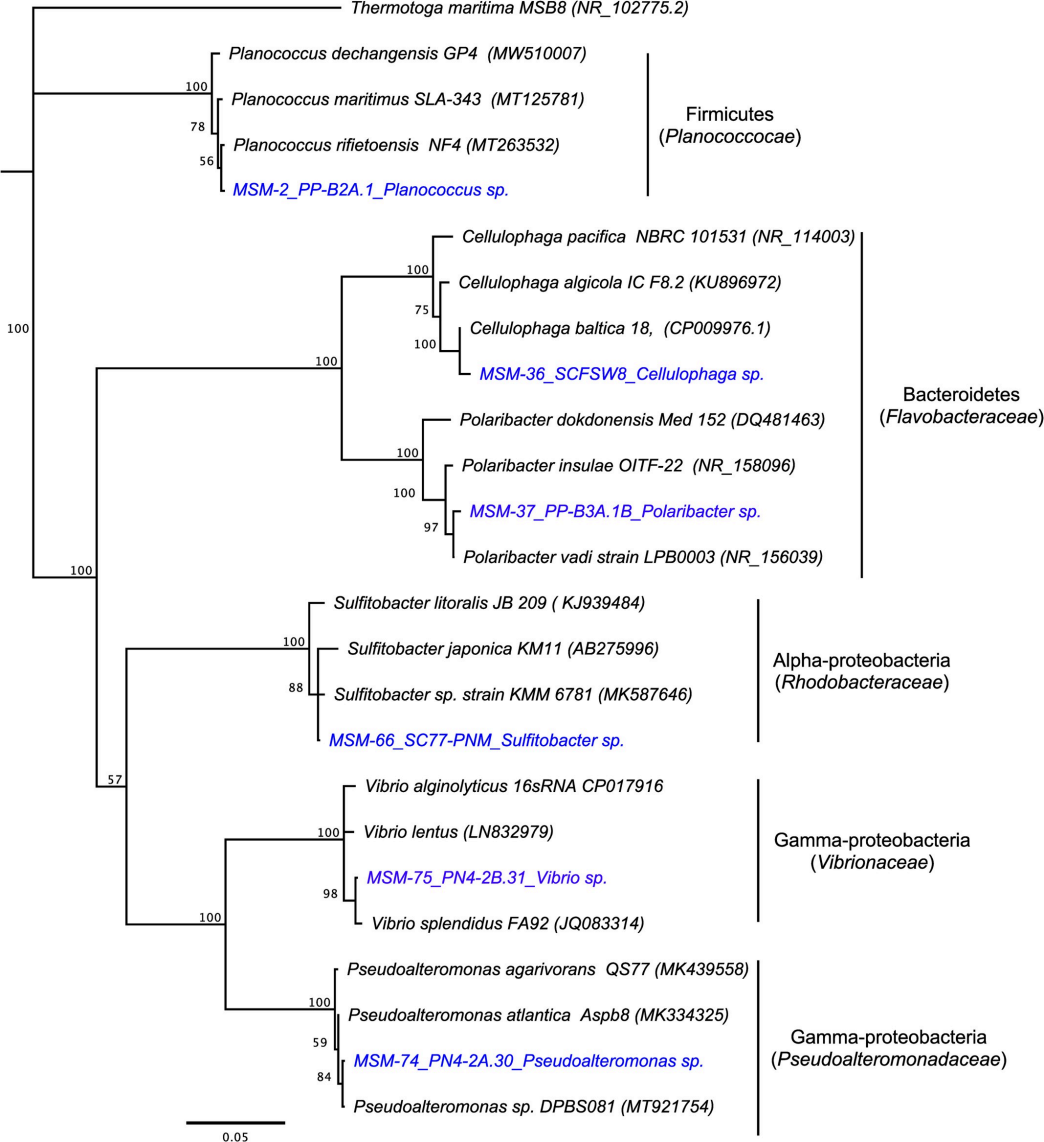
Phylogenetic tree of the bacterial isolates (blue color) in the study using 16S rRNA sequence as molecular marker. The tree was constructed using Neighbor-joining and Jukes-Cantor model for DNA evolution with 1000 bootstraps for tree resampling. Texts in parentheses are accession numbers of 16S rRNA homologous sequences downloaded from GenBank.

Marine bacteria used in this study were phylogenetically and morphologically distinct. *Cellulophaga* and *Polaribacter* are gram-negative bacteria members of the family *Flavobacteriaceae*, have similar elongated rod shapes, and produce yellow colonies in Difco Marine agar plates. The former was longer, having 4–5 μm in length (Fig 2A), while the latter was 2–2.5 μm in length (Fig 2B). *Planococcus*, a member of the Firmicutes phylum, is a gram-positive coccus-shaped bacterium (Fig 2C) with cells appearing as diplococci. Colonies appear as bright orange in agar media. The *Gammaproteobacteria*, *Pseudoalteromonas* sp. (Fig 2D), is a gram-negative, rod-shaped bacteria and about 1 μm in size. Membrane vesicles extend outward from their surface (Fig 2D). *Vibrio* sp. is a gram-negative, rod-shaped *Gammaproteobacteria* with varied sizes ranging from 1–2 μm (Fig 2E). Colonies of both *Gammaproteobacteria* appear white in agar plates. *Sulfitobacter*, a member of the family *Rhodobacteraceae* (SEM picture not available), produces small white colonies in agar plates. The phylogenetic affiliations of these bacteria based on their 16S rRNA sequences are shown in Fig 3.

### Algal growth rates with co-cultured bacteria

**Fig 4:**
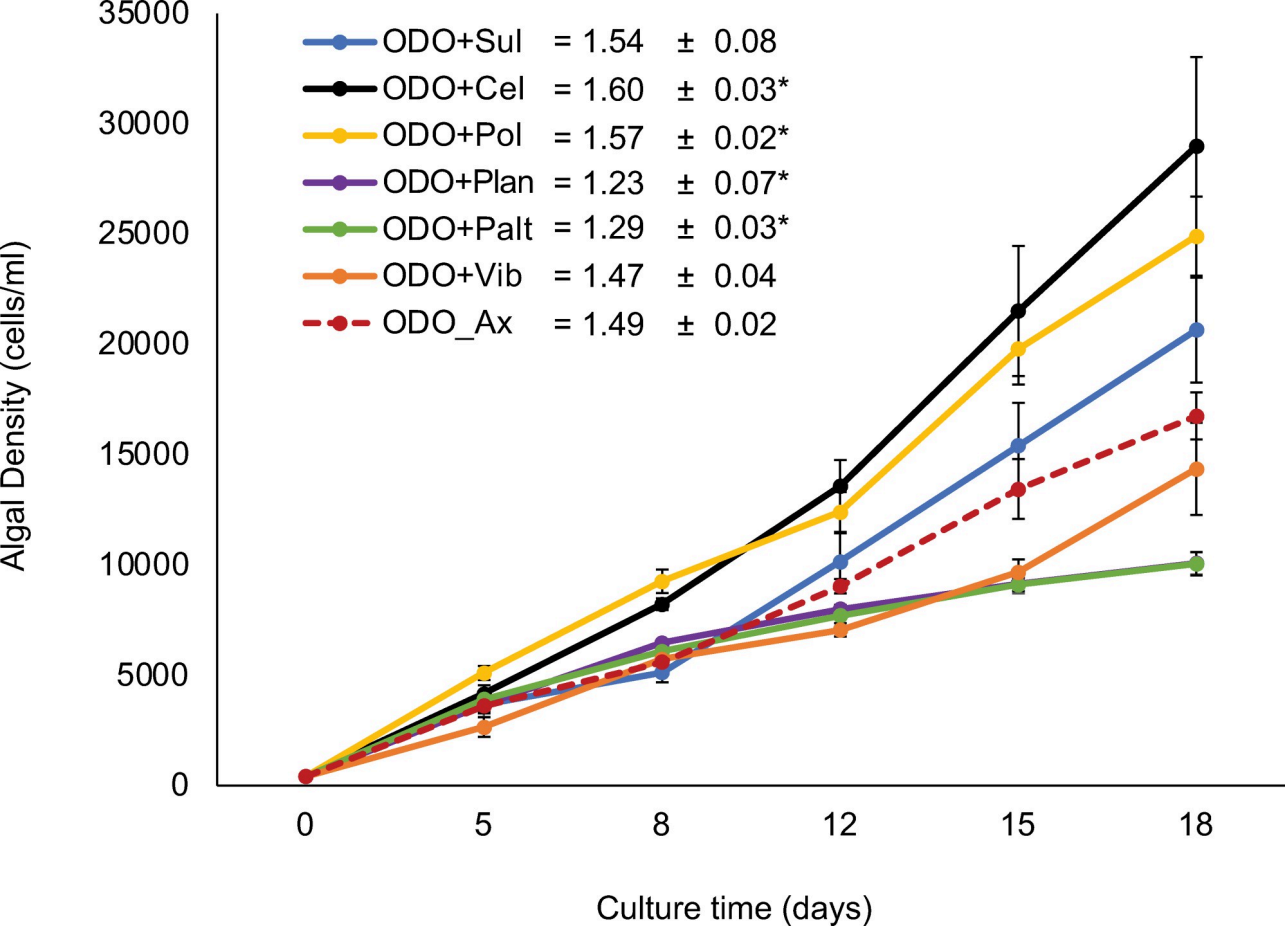
Algal densities and growth rates of *Odontella* sp. co-cultured with different bacterial isolates grown under the same culture conditions for 18 days. Growth rate was calculated between days 12 and 18, when most cultures displayed logarithmic growth stages. Algal densities (n = 4 for each treatment) at days 15 and 18 were log-transformed and analyzed using one-way ANOVA. Growth rates were analyzed using Kruskal-Wallis test, and means were compared using Wilcoxon pair test. * denotes a statistically different growth rate than axenic *Odontella* (ODO_Ax). Sul- *Sulfitobacter*; Cel- *Cellulophaga*; Pol- *Polaribacter*; Plan -*Planococcus*; Palt- *Pseudoalteromonas*; Vib- *Vibrio*; Ax- axenic.

Co-culturing *Odontella sp*. with different marine bacteria showed varying effects on diatom growth, especially when compared with the axenic *Odontella* sp. culture (Fig 4). Algal densities between treatments were significantly different at day 15 (F = 16.12, p = 0.0001) and at day 18 (F = 18.0611, p = 0.0001). Specific growth rates calculated at exponential growth (between days 12 and 18) also significantly vary between *Odontella* cultures (Kruskal Wallis test: χ^2^ = 20.9471, p = 0.0019). Co-culture with the Bacteroidetes bacteria *Cellulophaga* sp. and *Polaribacter* sp, achieved significantly higher algal cell densities at days 15 and 18 post-inoculation (Tukey-HSD, p < 0.05) and higher growth rates than axenic *Odontella* (Wilcoxon rank-sum test, p < 0.05). *Odontella* co-cultured with the *Gammaproteobacteria Pseudoalteromonas sp*. and the Firmicutes, *Planococcus sp*., showed significantly lower cell densities and growth rates than axenic *Odontella* culture (*Tukey-HSD*, p < 0.05). Interestingly, *Odontella* co-cultured with the *Gammaproteobacteria*, *Vibrio sp*., *and Alphaproteobacteria*, *Sulfitobacter sp*, showed no significant differences in algal growth with the bacteria-free *Odontella* (p > 0.05).

### Sexual cells and auxospore production

**Fig 5:**
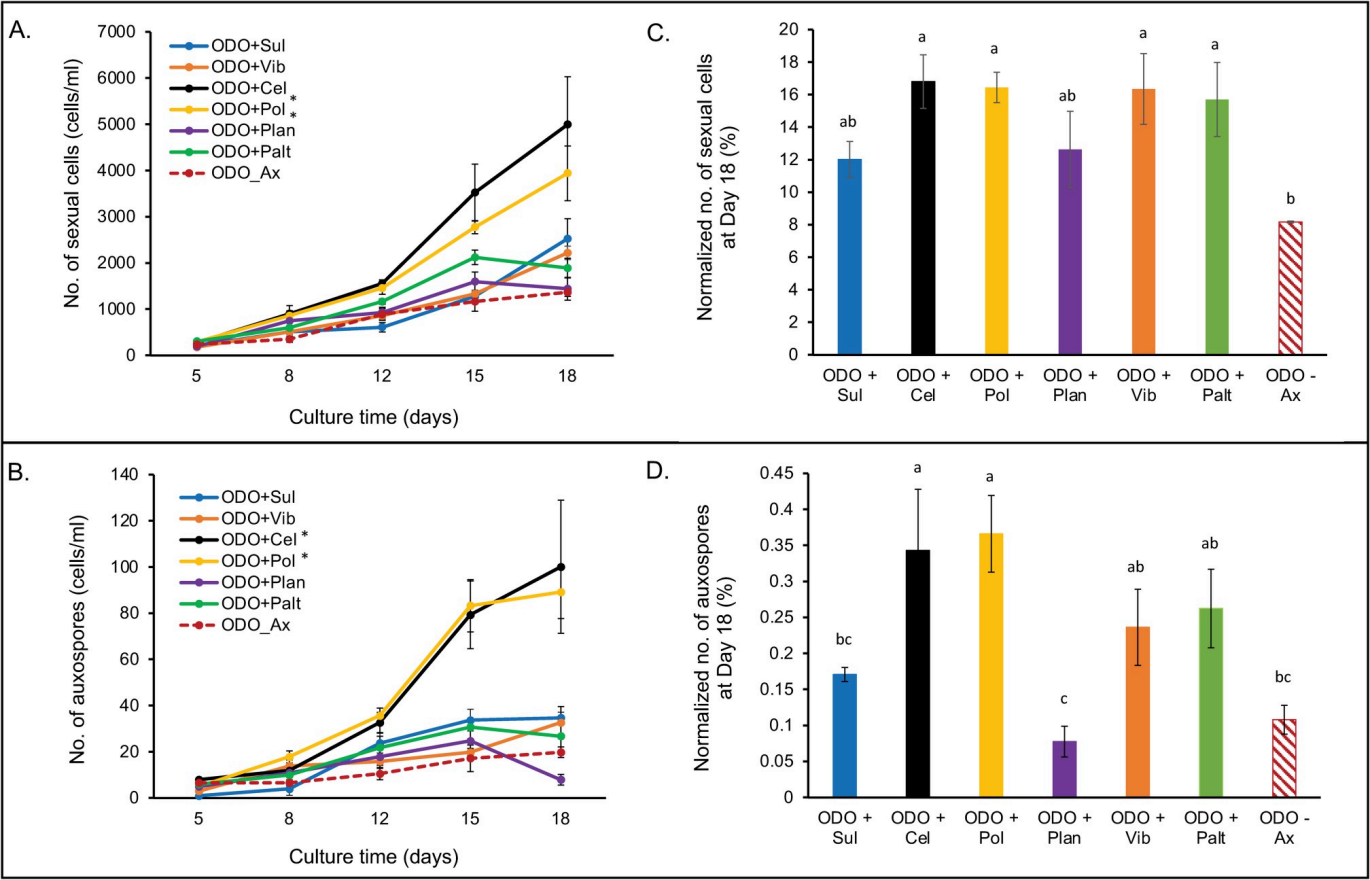
Differences in the number of sexual cells and auxospore production of *Odontella* sp. co-cultured with various bacterial isolates. **(**A) Number of sexual cells. (B) Number of auxospores. (C) Normalized number of sexual cells with total algal density. (D) Normalized number of auxospores with total algal density. Data (n = 4 for each treatment) were log-transformed (A and B) or arcsine-transformed (C and D) and were analyzed using one-way ANOVA followed by pairwise comparisons using Tukey-HSD. * denotes a statistically different number of sexual cells and auxospores than axenic *Odontella* (Odo_Ax). Column bars with the same letters denote no statistical difference.

Stages of *Odontella* sexual reproduction–plasmogamy, oocytes/egg cells, sperm cells, and auxospores (see images in Fig 1 and S1 and S2 Figs)–were tracked until day 18 and compared between treatments. These counts were calculated as the total number of sexual cells and auxospores (Fig 5A and 5B) and relative counts to total algal cell density (Fig 5C and 5D). An increase in the number of gametes and auxospores coincided with the exponential growth of the diatom. At the end of the experiment (day 18), *Odontella* cultures exhibited significant differences in the total number of sexual cells (F = 7.72, p = 0.0002) and the total number of auxospores (F = 14.85, p<0.0001). Notably, *Odontella* co-cultured with *Cellulophaga and Polaribacter* from the *Flavobacteriaceae* family exhibited a significantly higher total number of sexual cells and the total number of auxospores when compared to axenic *Odontella* and other co-cultures (Tukey-HSD, p<0.05) (Fig 5A and 5B). Interestingly at 18 days post-inoculation, axenic *Odontella* did not differ with *Odontella* co-cultured with *Vibrio*, *Sulfitobacter*, *Pseudoalteromonas*, or *Planococcus* with regards to total counts of sexual cells and auxospores (Tukey-HSD, p>0.05) (Fig 5A and 5B).

To further differentiate and compare the influence of bacteria on the sexual reproduction and auxosporulation of *Odontella* diatom, the total counts of sexual cells and auxospores were normalized with total algal density, and the ratios were expressed as percentages (Fig 5C and 5D). Significant differences in normalized sexual cells and auxospores between *Odontella* co-cultures were observed (F = 3.30 p = 0.02 and F = 5.97, p = 0.001, respectively). *Odontella* grown with *Cellulophaga*, *Polaribacter*, *Vibrio*, and *Pseudoalteromonas* had the highest ratios of sexual cells to total algal density and are significantly higher than axenic *Odontella (*Tukey-HSD, p<0.05) (Fig 5C and 5D). In contrast, the normalized number of sexual cells for *Odontella* co-cultured with *Planococcus* and *Sulfitobacter* did not significantly differ from the axenic *Odontella (*Tukey-HSD, p>0.05, Fig 5C). A slightly different pattern was observed with the normalized number of auxospores. Like the normalized number of sexual cells, the highest ratios of auxospore to total cell density were seen in co-cultures of *Odontella* with *Cellulophaga* and *Polaribacter*, and they were significantly different from the axenic *Odontella* (Tukey-HSD, p<0.05, Fig 5D). *Odontella* co-cultures with *Vibrio*, *Pseudoalteromonas*, and *Sulfitobacter* have statistically similar normalized number of auxospores with axenic *Odontella* (Tukey-HSD, p>0.05, Fig 5D). *Odontella* co-cultured with *Planococcus* had the lowest ratio of auxospores with total algal density and are significantly lower than those of *Odontella* co-cultured with *Flavobacteriaceae* and the *Gammaproteobacteria* bacterial isolates.

### Indole acetic acid production by marine bacteria

**Fig 6:**
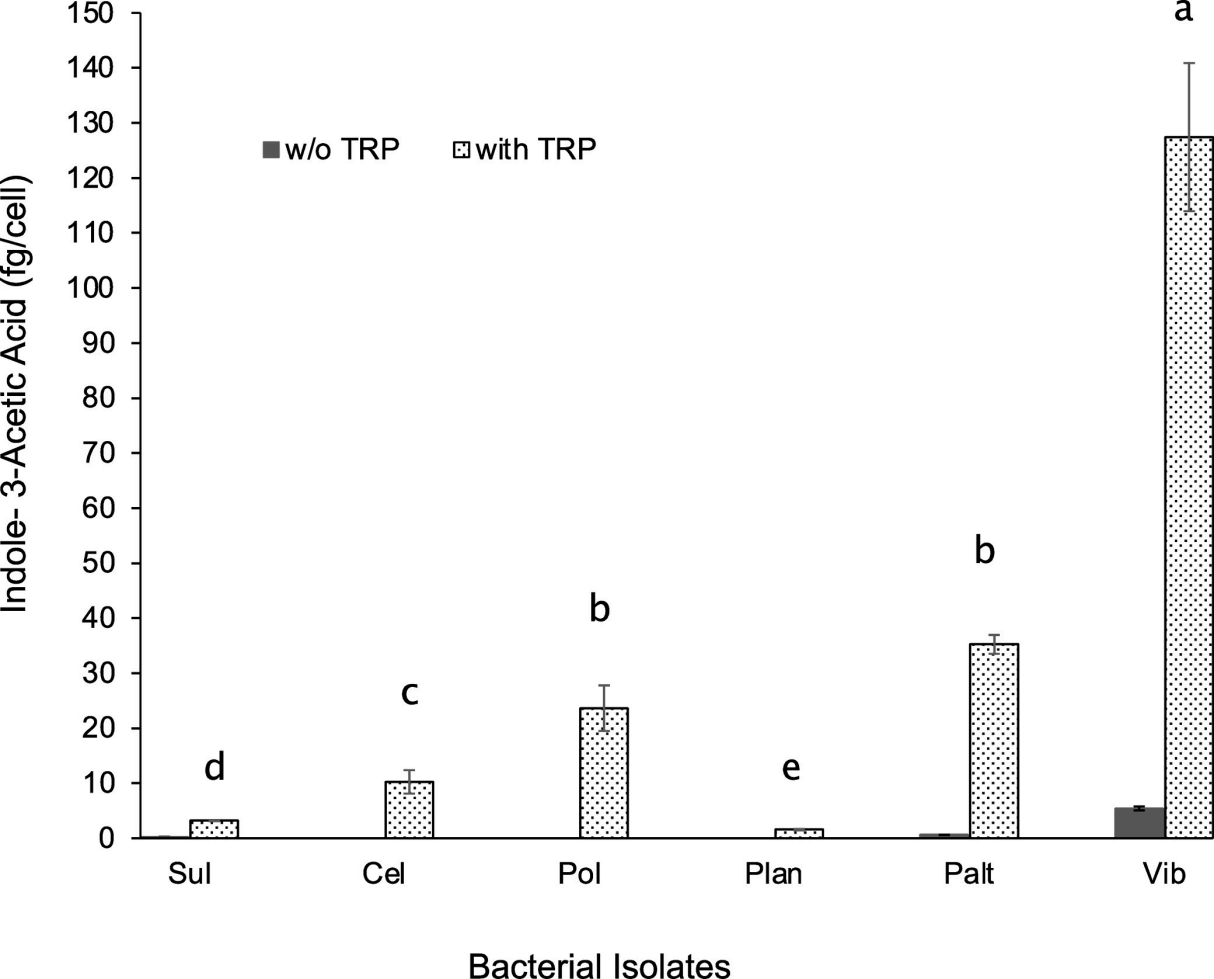
Indole acetic acid (IAA) secretion of bacterial isolates in the absence and presence of the IAA precursor tryptophan (TRP). Sul- *Sulfitobacter*; Cel- *Cellulophaga*; Pol- *Polaribacter*; Plan- *Planococcus*; Palt- *Pseudoalteromonas*; Vib- *Vibrio*. Data (n = 3 for each treatment) were log-transformed and analyzed using one-way ANOVA, followed by pairwise comparisons using Tukey-HSD. The letters above each column indicate statistically different IAA production (Tukey HSD, p<0.05).

The IAA metabolite secretion was measured and compared between bacterial isolates (Fig 6). Bacterial cultures were grown with and without tryptophan, and IAA was measured during bacterial exponential growth. Without tryptophan, IAA production was not detected for *Cellulophaga*, *Polaribacter*, and *Planococcus* and was minimally detected for *Sulfitobacter* (0.25 ± 0.03 fg/cell) and *Pseudoalteromonas* (0.61 ± 0.03 fg/cell). *Vibrio* sp. produced the highest IAA (5.23 ± 0.36 fg/cell) when grown without tryptophan. Adding tryptophan in the culture showed different trends in IAA production. In the presence of tryptophan, marine bacteria produced significantly different levels of IAA (F = 96.11, p<0.0001). *Planococcus* and *Sulfitobacter* bacterial strains had the lowest IAA production (Tukey HSD, p<0.005), while *Cellulophaga*, *Polaribacter* and *Pseudoalteromonas* significantly produced two to three times more IAA than the formerly mentioned bacterial strains in the presence of tryptophan ((Tukey HSD, p<0.005). Notably, the *Vibrio* sp. strain significantly produced about three to twenty times more IAA than the other bacterial strains (Tukey HSD, p<0.005).

### Growth and sexual reproduction of bacteria-free *Odontella* amended with IAA

**Fig 7:**
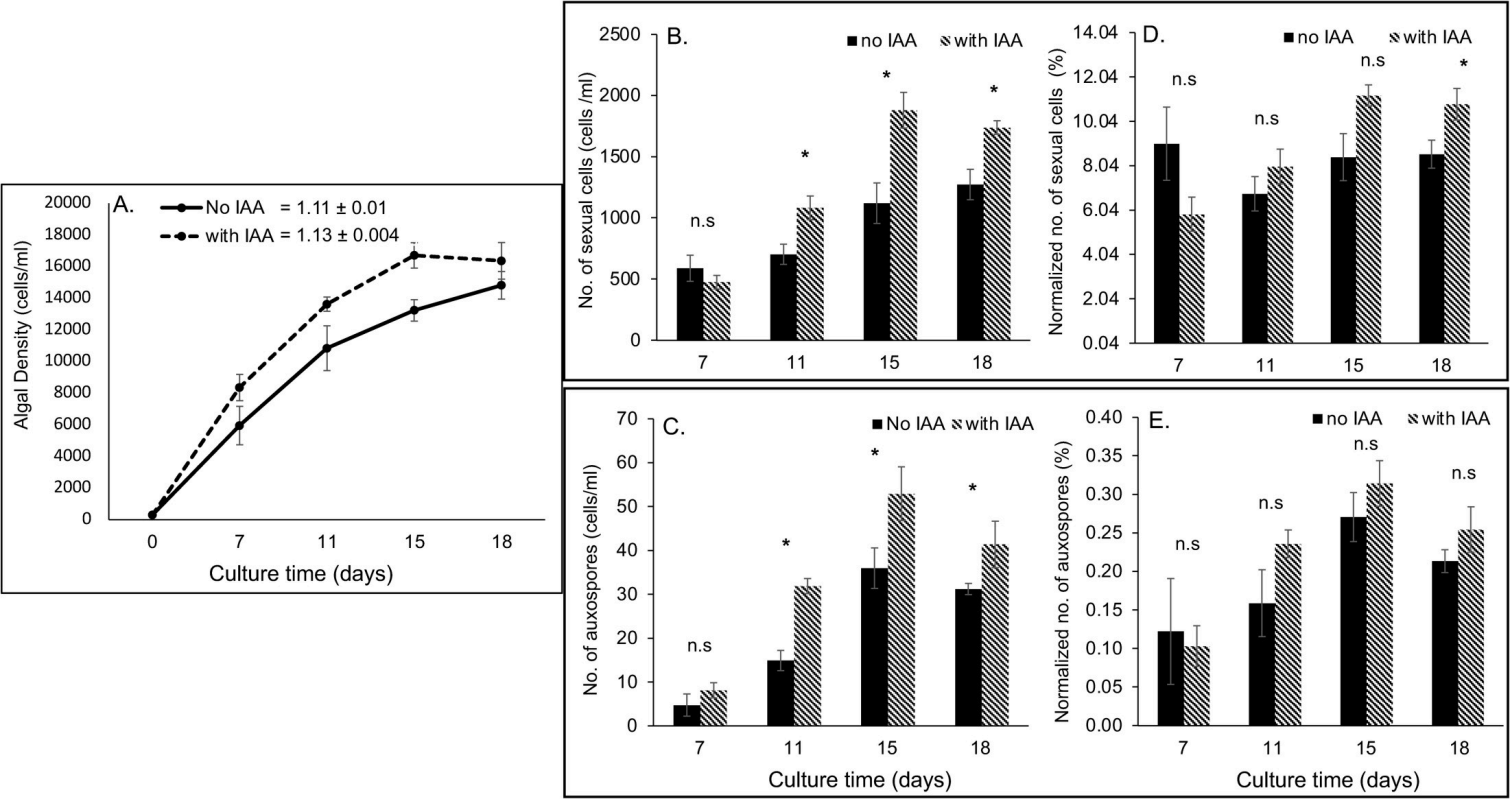
Growth and sexual reproduction of *Odontella* sp. cultures amended with and without IAA. **(**A) Algal densities (B) Number of sexual cells. (C) Number of auxospores (D) Normalized number of sexual cells. (E) Normalized number of auxospores. Growth rate (A) was calculated between days 7 and 15. Group means (B-E) were compared for each time point using Paired T-test (n = 5 for each treatment). * denotes a statistical significance at p< 0.05 while n.s. denotes no statistical difference.

To determine if IAA alone can influence the growth and sexual reproduction of axenic diatom, *Odontella* was grown with and without IAA and the number of sexual cells and auxospores was counted. Algal cell density of axenic *Odontella* amended with IAA was higher at the exponential growth stage (starting at day 7) than axenic *Odontella* without IAA. However, the growth rates of both cultures were not significantly different (Paired T-test, p>0.05, Fig 3A). Interestingly, the number of sexual cells and auxospores was significantly higher in IAA-amended *Odontella* cultures starting at day 11 (Fig 7B and 7C, Paired t-test, p<0.05). However, when these cells were normalized to total algal density for each sampling date, the percentage of sexual cells and auxospores produced were not significantly different between treatments (Fig 7D and 7E). Only on day 18 was a significant difference seen in normalized number of sexual cells between IAA-treated *Odontella* and untreated *Odontella*.

### Microbiome composition of *Odontella* cultures

**Fig 8:**
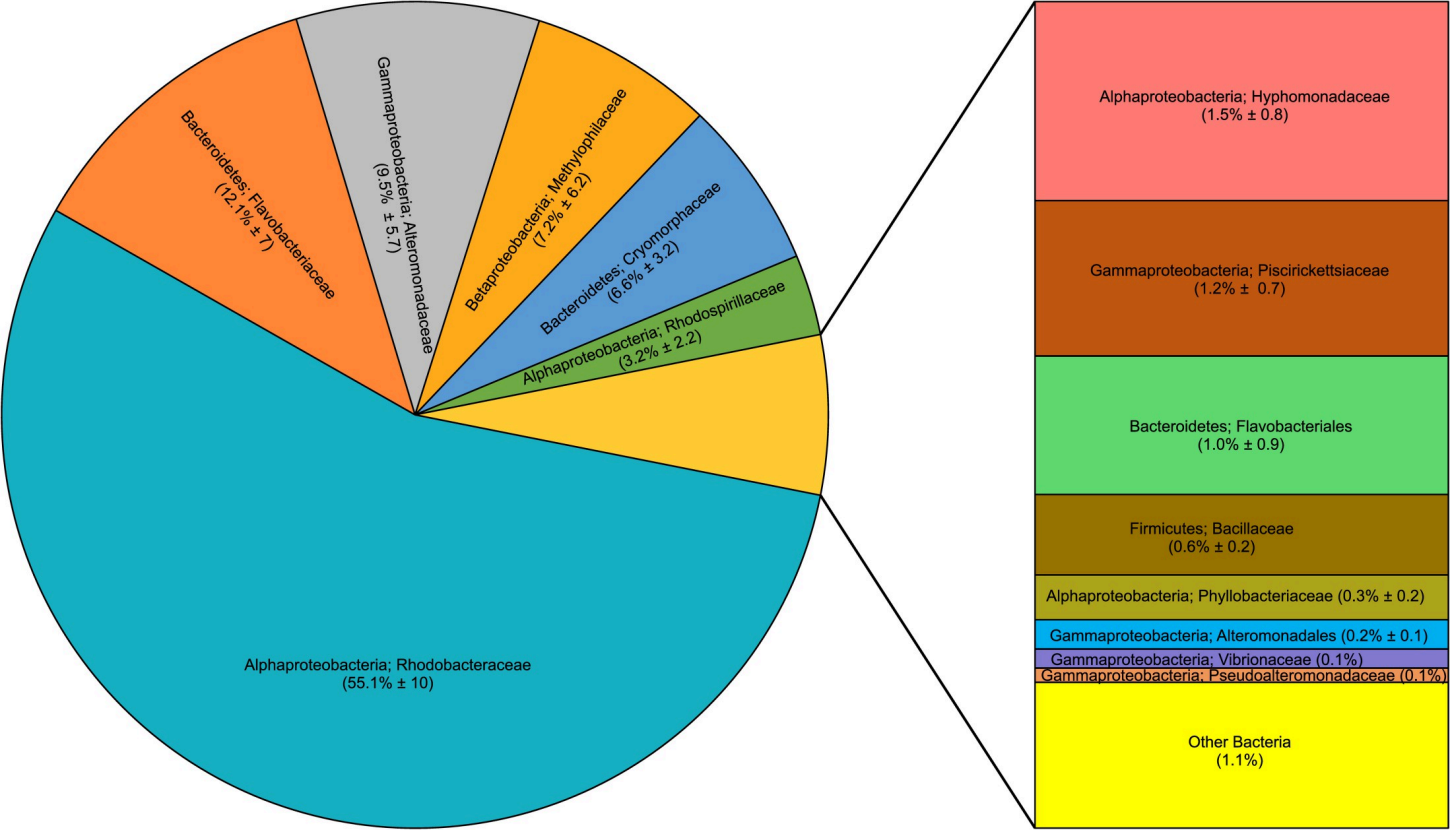
Microbiome community structure of *Odontella* sp. cultures using 16S rDNA amplicon sequencing. The relative abundance of OTUs summarized at the family level is shown. Microbiome members that are ≥0.1% (average relative abundance of n = 4) were included in the figure. Families with ≤0.1% were grouped as “Other Bacteria”. *Rhodobacteraceae*, to which *Sulfitobacter* is a member, and *Flavobacteriaceae*, to which *Polaribacter and Cellulophaga* are members, were the top two abundant families in the *Odontella* microbiome. *Vibrionaceae*, to which *Vibrio* is a member, and *Pseudoalteromonadaceae*, to which *Pseudoalteromonas* is a member, were rare in the microbiome assemblage. *Planococcoceae*, to which *Planococcus* is a member, was not detected in the *Odontella* microbiome.

To infer *Odontella*-microbiome partner association, we sequenced the microbiomes of four *Odontella* cultures. A total of 56,851 good-quality reads were generated, ranging from 6,583–25,889 reads per sample (n = 4). A total of 1195 OTUs were classified, but after removing the sequences of mitochondria, chloroplast, and singletons in the analysis, only 338 OTUS remained. These OTUs fell into groups of 4 phyla, 9 classes, 20 orders, 18 families, and 44 genera. Bacterial OTUs with ≥0.1% relative abundance were included in the tally (see S2 Table for relative abundance at the family level). The most abundant taxa were *Rhodobacteraceae* (55.1%±10), *Flavobacteraceae* (12.1%±7), and *Alteromonadaceae* (9.5%±6). Sequences homologous to the bacterial isolates *Sulfitobacter* (27%±5) and *Polaribacter* (6%±1) were abundant, while *Vibrio* and *Pseudoalteromonas* were very rare (≥0.1%) (Fig 8). Sequences homologous to bacterial isolates *Planococcus* and *Cellulophaga* were not detected.

### *Odontella* and microbiome co-occurrence in the coastal environment

**Fig 9:**
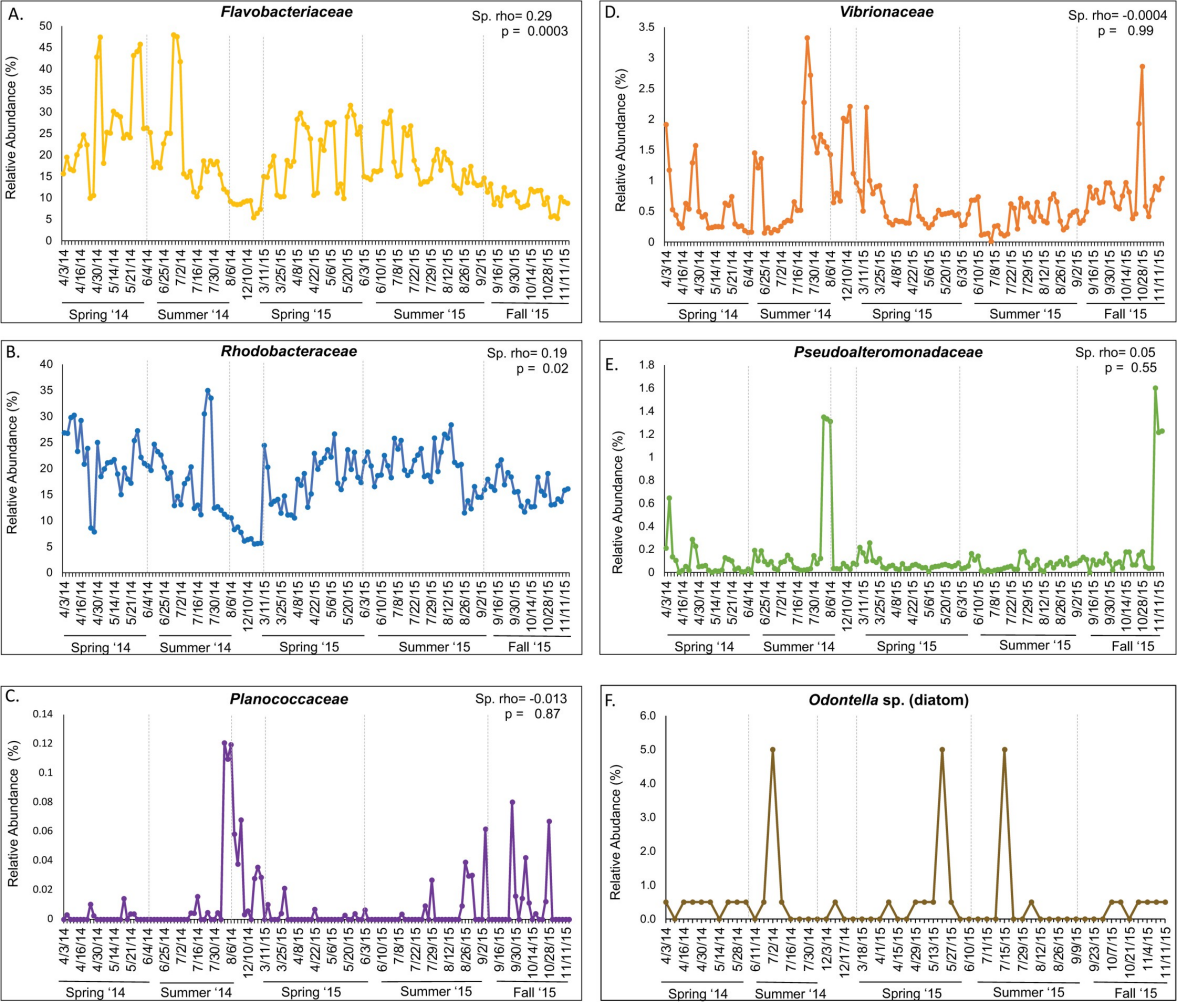
Relative abundance of the bacterial families and *Odontella* diatom at Santa Cruz Wharf, Monterey Bay, CA, surveyed between 2014–2015. (A) *Flavobacteriaceae* (B) *Rhodobacteraceae* (C) *Planococcaceae* (D) *Vibrionaceae* (E) *Pseudoalteromonadaceae* (F) *Odontella* sp. Bacterial abundances were determined via 16S rRNA amplicon sequencing, while *Odontella* diatom abundance was qualitatively surveyed using microscope counts. The strength of the association between the bacterial groups and the *Odontella* population was measured using Spearman rho correlation coefficient (Sp. rho) at p = 0.05.

To infer the co-occurrence of bacterial groups with *Odontella* diatom in a natural setting, we tracked the abundance of family *Flavobacteriaceae*, *Rhodobacteraceae*, *Pseudoalteromonadaceae*, *Vibrionaceae*, *Planococcaceae* using the available 16S rRNA sequences obtained from seawater samples in SC Wharf, Monterey Bay from 2014 to 2015. We found that the bacterial family *Flavobacteriaceae*, to which *Polaribacter* and *Cellulophaga* are members, was one of the most abundant bacterial families in 2014 and 2015 in SC Wharf water samples (Fig 9A). Both years showed the same trend; the highest abundance (up to 47.5% of the bacterial assemblage) was seen in spring and summer, and the population declined towards the Fall (5.3–6.3% of the bacterial assemblage). The *Rhodobacteraceae* family, to which *Sulfitobacter* is a member, was also abundant all year round (Fig 9B), with the highest abundance in spring and summer (up to 33.48%) followed by a slow decline during the Fall (5.6–11.4%). The *Planococcaceae* family, to which *Planococcus* is a member, was not detected in spring and early summer, but it was seen in the late summer of both years at a maximum abundance of 0.12% (Fig 9C). The *Vibrionaceae* family, to which *Vibrio* belongs, was present year-round but was not an abundant member of the bacterial assemblages. Its peaks were observed from mid-summer to Fall (2.8–3.3%, Fig 9D), while its lows averaged at 0.5%. This bacterial group had a higher abundance in 2014 than in 2015. The *Pseudoalteromonadaceae* family, to which *Pseudoalteromonas* is a member, was also not a dominant member of the bacterial assemblage, with peaks seen during mid-summer in 2014 and early Fall of 2015 (1.3–1.6%, Fig 9E). Its population is part of the background bacterial assemblage at an abundance range of 0.02% - 0.22%.

We also tracked *Odontella* diatom populations in the same two years (Fig 9F) to determine the potential co-occurrence of this diatom genus with these bacterial families. Unlike algal bloom-forming diatoms, *Odontella* is a minor member of the phytoplankton assemblage in SC Wharf, ranging from rare (0.5%) to present (5%). Furthermore, the diatom genus was seen in the phytoplankton assemblage during spring and summer, with zero to rare occurrences in the latter part of the summer and Fall (September to December) (Fig 9F). Interestingly, the bacterial populations belonging to *Flavobacteriaceae* and *Rhodobacteraceae* were significantly correlated with the presence of *Odontella* in the coastal site (Spearman rho = 0.29 and 0.19, p = 0.003 and 0.02, respectively), but not *Vibrionaceae*, *Pseudoalteromonadaceae*, or *Planococcaceae* (Spearman rho = -0.0004, 0.05, -0.013, respectively, p>0.05).

## Discussion

The microbiome is a non-genetic, environmental factor that is becoming recognized to play a role in the hosts’ morphological development and reproductive biology of eukaryotes. In this study, we asked if different phytoplankton-associated marine microbiota can influence the induction of sexual reproduction and auxosporulation in the ubiquitous marine diatom *Odontella* sp. Our study showed that bacteria-free *Odontella* has minimal production of sexual cells and auxospores, but sexual reproduction is enhanced in the presence of specific bacteria. Notably, bacteria from distinct phylogenetic groups exert varying degrees of influence on the clonal growth of the diatom host and the production of sexual cells and auxospores (Figs 4 and 5). Some bacteria are better promoters of clonal growth and sexual reproduction than others, such as the *Flavobacteriaceae* bacteria, *Polaribacter* sp. and *Cellulophaga sp*., which were consistently associated with more sexual cells and auxospores and a higher ratio of reproductive cells to total algal density. Interestingly, bacterial isolates from the *Vibrionaceae*, *Pseudoalteromonadaceae*, *Rhodobacteraceae*, *and Planococcoceae* family have no growth-promoting effect on *Odontella* as indicated by the slow accumulation of algal cells at day 18 (Fig 4). Interestingly, the respective effects of bacteria on the diatom’s production of gametes and auxospores are markedly different from their effects on asexual reproduction and growth. For instance, the Gammaproteobacterial isolates are associated with a higher proportion of sexual cells, but their diatom hosts grow slower than the bacteria-free cultures. In principle, diatom cells steadily undergoing vegetative division reach the critical minimum size faster than slow-dividing cells and are then triggered for sexual reproduction. However, the inconsistencies observed with *Vibrio* and *Pseudoalteromonas* co-cultures imply that factors other than the sexual size threshold may be involved in gametes and auxospore production, at least for *Odontella*. In this regard, we checked IAA secretion between bacteria to determine whether this metabolite is a potential trigger of sex in *Odontella*. IAA is a phytohormone belonging to a class of auxins known to promote the growth of terrestrial plants via root hair formation [43], cell elongation, and fruit development [44]. Auxins also act as morphogens that dictate cell division and gamete specification in terrestrial plants [45]. In microalgae, IAA produced by bacteria has been observed to promote the growth of *Emiliania huxleyi* [46], *Pseudo-nitzschia multiseries* [23] and *Chlorella pyrenoidesa* [47]. IAA synthesis is triggered by tryptophan, an IAA precursor released by marine algae [46, 48]. Indeed, IAA secretions vary between bacterial isolates, with *Vibrio*, *Pseudoalteromonas*, *Polaribacter*, *and Cellulophaga* showing higher secretions in the presence of tryptophan (Fig 6). These four bacteria also showed the maximal influence on the production of gametes, despite showing contrasting effects on diatom growth. *Vibrio and Pseudoalteromonas*, for example, can stimulate the production of sexual cells in the diatom host, despite not being clonal growth promoters. Notably, these two bacteria have the highest IAA production. These results suggest that the modulation of clonal growth and sexual reproduction in *Odontella* is possibly influenced by separate mechanisms other than IAA.

Indeed, we verified the influence of IAA on diatom growth and sexual reproduction by amending axenic cultures with synthetic IAA. The amendment of synthetic IAA (50nM) to bacteria-free *Odontella* only slightly improved clonal growth but increased the number of sexual cells and auxospores as the bacteria-free diatom cells grew exponentially (Fig 7B and 7C). However, the percentage of gametes and auxospores in both populations is comparable. This suggests that synthetic IAA can influence diatom growth and gamete and auxospore production, but the effect is nominal (Fig 7). Moreover, this implies that sexual reproduction is possibly stimulated by other factors, perhaps by another bacterial metabolite other than IAA. Indeed, different forms of auxins [i.e., indole-3-acetic acid (IAA), phenylacetic acid (PAA), and indole-3-butyric acid (IBA)] have been described, which could be synthesized via tryptophan-dependent and tryptophan-independent biosynthetic routes [49, 50]. The auxin, phenylacetic acid, for instance, is secreted by *Phaeobacter gallaeciensis*, a *Roseobacteraceae* bacteria, that promotes the growth of the bloom-forming coccolithophore *Emiliania huxleyi* [51]. Cytokinin-active compounds are also morphogens that could promote growth, development, cell division, and delay of senescence in plants [52]. The cytokinin compound isopentenyladenine has been detected in seawater and was reported to promote the growth of axenic brown seaweeds [53]. The same cytokinin compound is produced and secreted by several marine bacteria, including *Vibrio*, *Flavobacterium*, and *Alteromonas* [54, 55]. Interestingly, the production of cytokinin and auxin compounds by different marine bacteria could vary in amount, depending on where these bacteria were found (i.e., seaweeds, phytoplankton, sediments, or seawater) [55]. Measuring cytokinins and other auxin metabolites secreted by bacterial associates would be an interesting follow-up investigation to our present study.

The production of auxospores is a unique feature in diatoms because it can restore the diatom’s diminishing cell size, although another method, such as cell size enlargement, has been reported [56, 57]. The successful induction of sex in diatoms go through a two-stage process; first, the production of gametes which is mainly triggered upon reaching the sexual size threshold; second, the successful meeting and fusion of gamete partners to form the auxospore. Environmental and chemical cues likely assert their influence on the gamete production stage. For instance, low light level, short photoperiod, and exposure to red light spectra have been reported to influence the production of gametes and auxospores in *Haslea ostrearia* [12]. In *Leptocylindricus danicus*, sexual reproduction can be induced under nitrate-depleted conditions and at 16°C [58, 59], while in *Skeletonema marinoi*, changes in the salinity of 6 to 16 psu can induce sexualization [60]. Short photoperiod can accelerate the auxospore formation of *Coscinodiscus concinnus* [61], while a high ammonia level can induce sexual reproduction in *Thalassiosira pseudonana* [11]. Our study did not use any special procedure to induce sexual reproduction in *Odontella*, and the diatom cells were initially grown in nutrient-replete conditions under constant temperature and photoperiod. Sexual cells and auxospores were observed beginning at day 5 and were maximally counted near the end of the exponential stage (days 15 to 18), where nutrients are most likely starting to deplete and when cells have reached a high density (Fig 4). After gametogenesis, a pheromone signal likely guides the pairing of gametes. Sexual cells in pennate diatoms release pheromones to sense partners [9, 62]. Interestingly, the pheromone production by the benthic pennate diatom *Seminavis robusta* is modulated by the associated bacteria; this, in turn, increases gamete pairing, resulting in more auxospores [13, 14]. It would be interesting to determine if a similar mechanism explains the enhancement of auxospore production in *Odontella* by the *Flavobacteriaceae* bacteria. However, whether sexual cells in centric diatoms use pheromone or chemoattractant for partner signaling is unknown.

Without bacteria, *Odontella* can still produce gametes and auxospores (8% and 0.1%, respectively) under sufficient nutrients, temperature, and light conditions. In the presence of sex-enhancing bacteria such as the *Flavobacteriaceae*, gamete and auxospore production increased two- or three-fold (17% and 0.37%, respectively), suggesting that the associated bacteria have a positive influence on *Odontella*’s reproductive biology. *Odontella* is not known to maintain an obligate symbiosis with bacteria; however, our microbiome survey from *Odontella* cultures points to long term, stable association of *the* diatom with some of these bacteria, especially *Flavobacteriaceae* and *Rhodobacteracea*e (Fig 8), even after 130 culture generations. Recent literature suggests that diatom-associated bacteria are not randomly assembled but selected based on the diatom host traits, whose association is stable over time [63, 64]. The *Odontella* cultures in our study were isolated and cultured from water samples when *Flavobacteriaceae* and *Rhodobacteraceae* are the two most abundant microbiome members (see Fig 9). These bacteria were most likely isolated together with the *Odontella* at the time of culture. After 130 culture generations, *Flavobacteriaceae* and *Rhodobacteraceae* were still the dominant microbiome members in these *Odontella* cultures. We can only surmise that their partnership over several generations in culture has been sustained via the mutualistic exchange of metabolic products. Phytoplankton tends to leak some of their photosynthates; some are considerable amounts of polysaccharides that accumulate extracellularly around the cells [65], which some bacteria like the *Flavobacteriaceae* members, are genetically capable of utilizing [66, 67]. The abundance of *Flavobacteriaceae* bacteria has also been observed to peak during diatom blooms [19, 66], indicating that these bacterial types interact in nature with various marine diatoms. The abundance of *Flavobacteriaceae* also correlates with the *Odontella* diatom in Monterey Bay (Fig 9), suggesting that *Odontella*-*Flavobacteriaceae* association is not random.

Some bacteria are not as mutualistic as others despite being phytoplankton-associated. The *Rhodobacteraceae* bacteria, *Sulfitobacter*, for example, had no growth- and sex-promoting effect on *Odontella* despite being the dominant microbiome in culture and co-occurring naturally with the *Odontella* population. *Rhodobacteraceae* group tends to be one of the dominant members of the bacterial microbiome during phytoplankton blooms [68, 69], and many *Sulfitobacter* strains are known to associate and promote the growth of different marine diatom genera [23, 70, 71]. Compared to other bacterial isolates in the study, IAA production of *Sulfitobacter* is minimal, which may explain its negligible effect on *Odontella* growth and sexual reproduction. On the other hand, the *Planococcaceae* bacteria, *Planococcus*, depressed the clonal growth of *Odontella* and showed no influence on the diatom’s sexual reproduction. *Planococcus* also showed minimal IAA secretion. *Planococcus* are Firmicutes bacteria that peak near the end of a diatom bloom [18], hinting that the bacteria are particle degraders. Species members of this genus are known to release biosurfactants and metabolites that degrade various types of hydrocarbons [72]. Interestingly, *Planococcus* was not found in the microbiome assemblage of cultured *Odontella*, and it did not co-occur with *Odontella* populations in our 2-year ocean microbiome survey.

Our study has demonstrated that some specific bacteria strongly influence the successful expression of a distinct evolutionary trait in a marine diatom, promoting clonal growth, maintaining genetic diversity, and restoring diatom cell size through sexual reproduction. Associated bacteria can modulate an essential biological process in marine diatoms in the form of sex, facilitating the evolutionary and ecological success of these important primary producers. The results in this study lend strong support to the emerging concept that diatom-bacteria interaction is not based on random chance but is instead shaped strongly by evolution and partner compatibility leading to increase diatom fitness. Our results also have substantial biotechnological implications; sex-enhancing bacteria can be utilized for the long-term culture maintenance and breeding of *Odontella* and for improving the genetic manipulations of its desirable traits for health and cosmetic industry applications.

## Supporting information

S1 Fig Additional spermatogonia images of *Odontella*. The diatom cell elongates during spermatogenesis, with spermatocytes seen inside the cells. An opening in the frustule facilitates the release of sperms (arrows).
(PDF)

S2 Fig Additional eggs and auxospores images of *Odontella*. One or two eggs are produced by a diatom cell and are released during plasmolysis (A-F). Auxospores at various stages of development, with the distinct perizonium (arrows) visible for each auxospore (G-L).
(PDF)

S1 Video Sperm tail moving near the distal end of the cell. (MOV)

S1 Table BioSample IDs of 16S rRNA sequences submitted in SRA database. (TXT)

S2 Table Relative abundance of *Odontella* microbiome at the family level. (TXT)

S1 File Centered log-ratio transformation of OTU abundance data in R. (PDF)
