# Supplementary figures and images for: Specific bacterial microbiome enhances the sexual reproduction and auxospore production of the marine diatom, Odontella
Source: PLoS One. 2022 Oct 19;17(10):e0276305. doi: 10.1371/journal.pone.0276305 (PMC9581435; doi:10.1371/journal.pone.0276305)

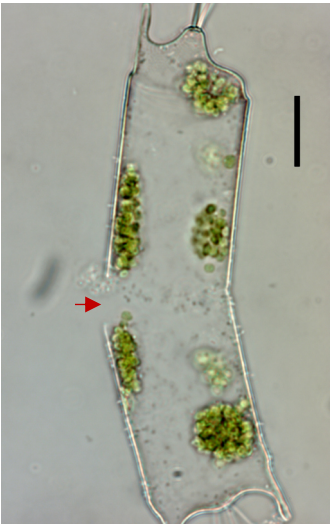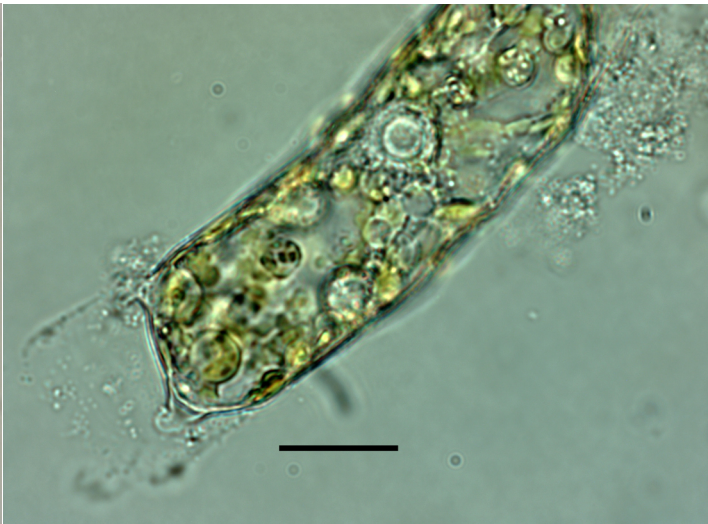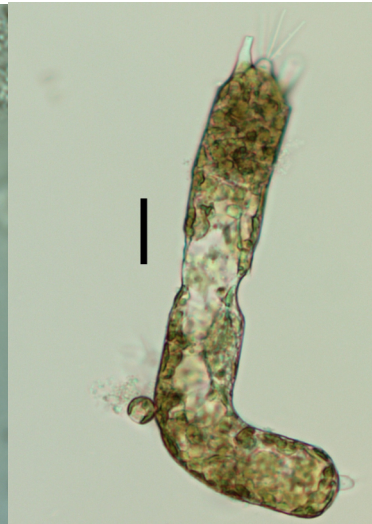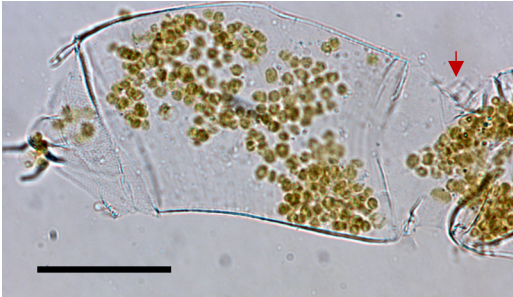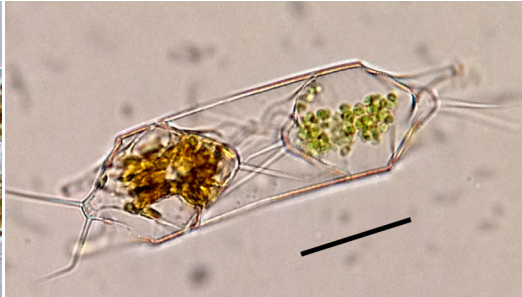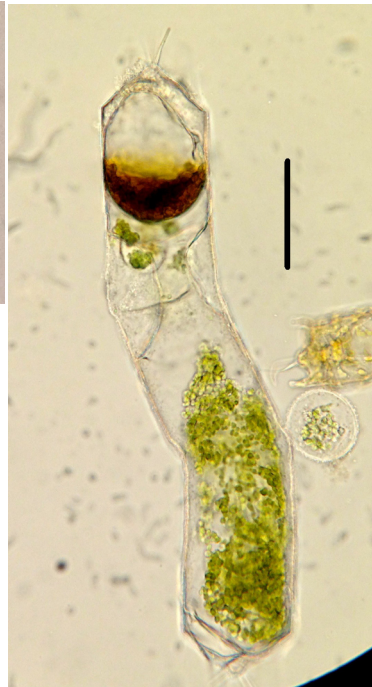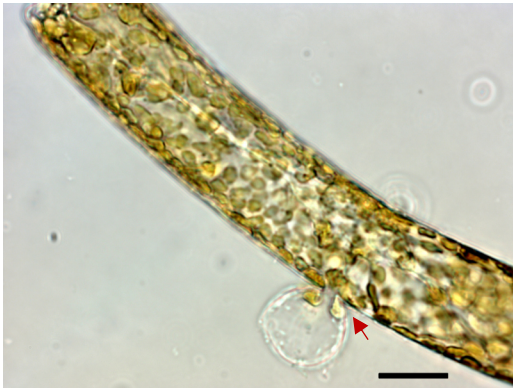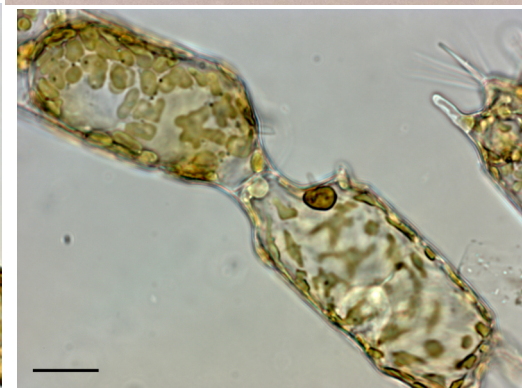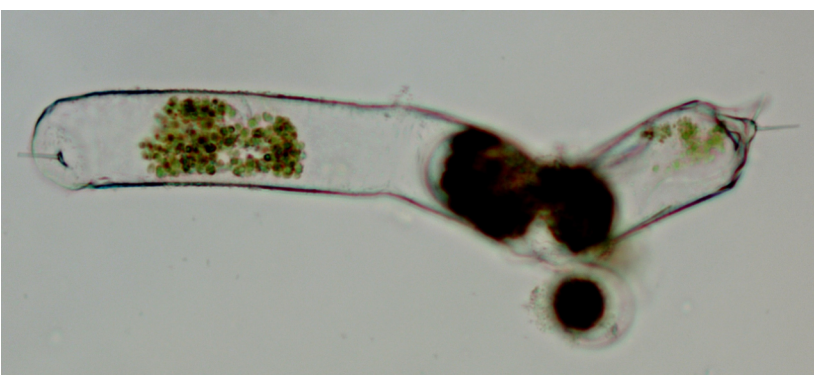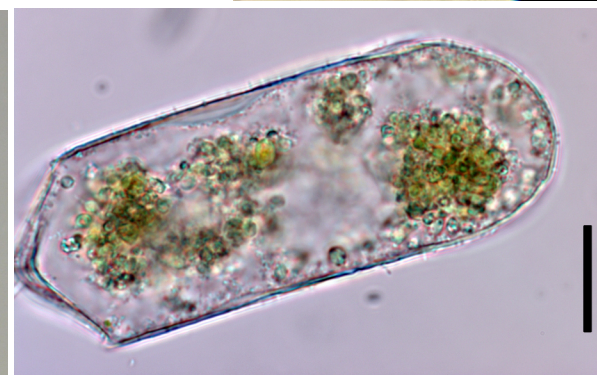

Supplement: S1 Fig — The diatom cell elongates during spermatogenesis, with spermatocytes seen inside the cells. An opening in the frustule facilitates the release of sperms (arrows). (PDF) [file pone.0276305.s001.pdf]

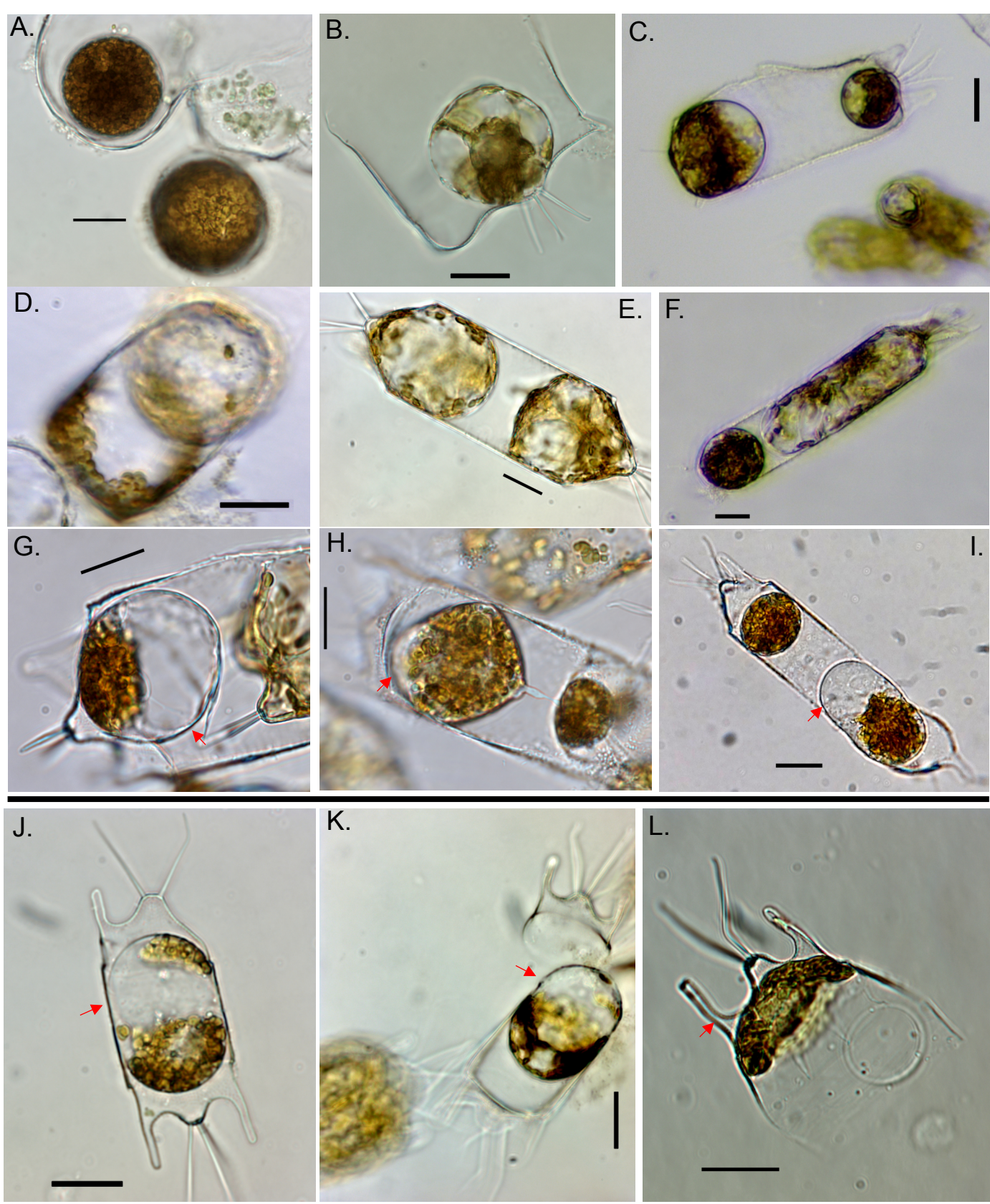

Supplement: S2 Fig — One or two eggs are produced by a diatom cell and are released during plasmolysis (A-F). Auxospores at various stages of development, with the distinct perizonium (arrows) visible for each auxospore (G-L). (PDF) [file pone.0276305.s002.pdf]
