# Supplementary material for: Specific bacterial microbiome enhances the sexual reproduction and auxospore production of the marine diatom, Odontella
Source: PLoS One. 2022 Oct 19;17(10):e0276305. doi: 10.1371/journal.pone.0276305 (PMC9581435; doi:10.1371/journal.pone.0276305)
Supplement: S1 File — (PDF) [file pone.0276305.s006.pdf]

```
library(compositions)

library(zCompositions)


#The first portion of this program takes in binned OTU count data (file input line 7).

#Then removes 0s by substituting a single count and maintaining the proportion of the rest of the originally non-0 values.

#Then Centered Log-Ratio transforms the data for later use in PCA-type analyses, etc.

#Inputs required are a trimmed OTU count file in .csv with columns as sample number and rows as OTUs,

#and optionally a file of dates/sample info to be appended to the dataframe before output also in .csv with rows as sample numbers and columns as data fields.


#Import the sample table

count_table<-read.csv("30um_counts_trimmed50.csv",row.names=1,check.names=FALSE)

#Transpose the data to have sample names on rows

count_table<-t(count_table)


#transform zeros to small positive numbers while maintaining ratios between non-zero components. Necessary for CoDA. Converts absolute counts to relative abundance in the process.

abund_table_no_0<-cmultRepl(count_table)


#CLR transformation

clr_abund_table<-as.data.frame(clr(abund_table_no_0))


#Import date labels, append to transformed relative abundance data frame. This step is optional and can be commented out.
```

```
dateid<-read.csv("sample_metadata.csv", row.names = 1)

clr_abund_table<-merge(x=dateid, y=clr_abund_table, by="row.names", all.y = TRUE)

clr_abund_table<-subset(clr_abund_table, Row.names != "Sum")

clr_abund_table<-data.frame(clr_abund_table, row.names = 1)



#Write output to .csv file

write.csv(clr_abund_table, "30um_abund_CLR.csv")



#Subset data to only specific sequence ids.

#The file is currently required to have extra rows matching any metadata that should be carried over, in this case Date, pore size and replicate.

seqsofint<-read.csv("seq_ids.csv", row.names=1)

clr_abundt<-t(clr_abund_table)

#Merge the datasets keeping only columns that appear in both, then set row names for the new database

clr_soi <- merge (x=seqsofint, y=clr_abundt, by="row.names")

clr_soi <-data.frame(clr_soi, row.names=1)

#save to a .csv file - This is the readable one

write.csv(clr_soi, "30um_CLR.csv")


# At this point the data is ready for analysis. You can use the CSV on its own or continue below to output timeseries data
```
